# Draft Genome Sequence of Bacillus urumqiensis BZ-SZ-XJ18^T^, a Moderately Haloalkaliphilic Bacterium Isolated from a Saline-Alkaline Lake

**DOI:** 10.1128/genomeA.00460-18

**Published:** 2018-05-31

**Authors:** Ziya Liao, Chao Ren, Xiaomeng Guo, Yanchun Yan, Jun Li, Baisuo Zhao

**Affiliations:** a Graduate School, Chinese Academy of Agricultural Sciences, Beijing, People’s Republic of China; b Institute of Agricultural Resources and Regional Planning, Chinese Academy of Agricultural Sciences, Beijing, People’s Republic of China

## Abstract

The moderately haloalkaliphilic bacterium Bacillus urumqiensis BZ-SZ-XJ18^T^ was isolated from a saline-alkaline lake located in the Xinjiang Uyghur Autonomous Region of China. Optimum growth occurred at the total Na^+^ concentration of 1.08 M, with a broad optimum pH of 8.5 to 9.5. The draft genome consists of approximately 3.28 Mb and contains 3,228 predicted genes. A number of genes associated with adaptation strategies for osmotic balance and alkaline pH homeostasis were identified, providing pertinent insight into specific adaptations to the double-extreme environment.

## GENOME ANNOUNCEMENT

The moderately haloalkaliphilic bacterium Bacillus urumqiensis BZ-SZ-XJ18^T^ was isolated from a saline-alkaline lake in the Xinjiang Uyghur Autonomous Region of China (1). Its growth occurs at a total Na^+^ concentration of 0.22 to 4.32 M (optimum concentration, 1.08 M Na^+^) and at pH 6.5 to 10.0 (optimum pH, 8.5 to 9.5). To understand the adaptive strategies for survival under saline-alkaline conditions, draft genome sequencing of strain BZ-SZ-XJ18^T^ was performed using the Illumina Miseq sequencing platform.

Genomic DNA was extracted using an iTop microbial DNA isolation kit (Beijing, China) following the manufacturer's instructions. A library for genome sequencing was constructed using the whole-genome shotgun (WGS) approach on the Illumina platform (2). Sequencing was performed with a paired-end read length of 2 × 300 bp at approximately 382× coverage. Genomic contigs were *de novo* assembled though the A5-miseq v 20150522 pipeline (3). Quake and Burrows-Wheeler Aligner (BWA) were used to detect and correct errors in preassembly and postassembly sequences (4, 5). The total length of the draft genome sequence was 3,280,710 bp, and there were 26 contigs, with a GC content of 51.7% and an *N*_50_ value of 219,019 bp. Automated gene annotation was obtained using the NCBI Prokaryotic Genome Annotation Pipeline (www.ncbi.nlm.nih.gov/genome/annotation_prok). Subsequently, the genome files in GenBank format (gb file) were uploaded to the Integrated Microbial Genomes Expert Review (IMG ER) tool (https://img.jgi.doe.gov/cgi-bin/submit/main.cgi) for functional annotation after an Analysis Project identification number (ID) was registered in the GOLD Database (https://gold.jgi.doe.gov/index). Among the identified 3,228 genes, there are 3,157 putative protein-coding sequences (CDSs). A total of 71 RNA genes (4 5S rRNAs, 1 16S rRNAs, 3 23S rRNAs, 59 tRNAs, and 4 other RNA genes) were predicted.

The genome sequence revealed the presence of a number of genes encoding putative proteins potentially related to the strategies for surviving in double-extreme conditions of elevated salinity and alkaline pH. Two *ectA* genes, 2 *ectB* genes, and 1 *ectC* gene for ectoine synthesis, 1 *glnA* gene for l-glutamine synthesis, and 1 *speE* gene for spermine synthesis were identified. Two genes for glycine betaine synthesis from choline, 6 genes for the glycine betaine-carnitine-choline transporter (BCCT) family, and 1 gene encoding the sodium/proline symporter of the solute-sodium symporter (SSS) family were found. These demonstrate a mechanism, a means of coping with hyperosmotic conditions (higher external salinity), by amassing large quantities of compatible solutes, including ectoine, glutamine, spermine, and glycine betaine (6–9). The presence of 3 *trkA* genes and 2 *trkH* genes, which participate in the Trk system for potassium uptake protein synthesis, indicated that strain BZ-SZ-XJ18^T^ also maintains osmotic balance by inducing a massive uptake of K^+^ in high-salinity environments (10). As a haloalkaliphile, B. urumqiensis must have an adaptive strategy for both salinity homeostasis and pH homeostasis. The genome harbors 2 genes for calcium/proton antiporters of the Ca^2+^ calcium antiporter (CaCA) family, 1 gene encoding potassium/proton antiporters of the CPA1 family, 1 gene for the sodium/proton antiporter of the CPA1 family, 7 multisubunit sodium/proton antiporters, 10 genes for F_1_F_o_-ATP synthase, and 1 gene encoding a sodium/proton antiporter of the NhaC family deployed for alkaline pH homeostasis (11). As described in this report, various predicted genes on the genome of B. urumqiensis offer valuable insights to reveal the adaptive mechanisms of this haloalkaliphile.

### Accession number(s).

The draft genome sequence of B. urumqiensis BZ-SZ-XJ18^T^ has been deposited at DDBJ/ENA/GenBank under the accession number PVNS00000000.
